# Construction of an ATP hydrolysis-related 11-gene signature for predicting prognosis and immune response in hepatocellular carcinoma

**DOI:** 10.1097/MD.0000000000050860

**Published:** 2026-09-25

**Authors:** Yan Dong, Feng Yu

**Affiliations:** a Department of Digestive Internal Medicine, No. 906 Hospital of the People’s Liberation Army, Ningbo, Zhejiang, China.

**Keywords:** ATP hydrolysis, hepatocellular carcinoma, immune landscapes, prognosis, treatment responsiveness

## Abstract

The dysregulation of adenosine triphosphate (ATP) hydrolysis, a crucial process in energy metabolism, has been implicated in the complex landscape of tumor development and progression. This study aims to pinpoint crucial genes that characterize the ATP hydrolysis-driven molecular signature of hepatocellular carcinoma (HCC), with a view to examining their potential therapeutic applications in enhancing patient prognosis. Utilizing transcriptomic and clinical data from The Cancer Genome Atlas and Gene Expression Omnibus databases, a systematic framework was developed and validated for model construction and assessment. Univariate Cox regression analysis (*P* < .05) was performed on all 416 ATP hydrolysis-related genes (ARGs) to identify prognostically relevant genes. Following least absolute shrinkage and selection operator Cox regression with 10-fold cross-validation, an 11-gene signature was established, with the median training cohort risk score used as the cutoff for stratifying patients into high- and low-risk groups. Among 416 investigated ARGs, 85 displayed differential expression in HCC, with 179 demonstrating significant prognostic relevance (univariate Cox *P* < .05). Two distinct molecular subtypes, characterized by marked differences in prognosis and immune infiltration, were identified through unsupervised clustering. A refined 11-gene signature was constructed via least absolute shrinkage and selection operator Cox regression with 10-fold cross-validation (training area under the curve: 0.826/0.717/0.657 for 1/3/5 year; validation area under the curve: 0.608/0.603/0.621). These risk groups exhibited significantly divergent prognostic features, gene expression patterns, immune microenvironments, and drug sensitivities, suggesting the utility of this risk signature in predicting prognosis, drug responsiveness, and immune therapeutic outcomes. Ultimately, a nomogram incorporating the risk signature was devised, demonstrating favorable predictive performance in estimating HCC patient prognosis compared to other established prognostic factors. This study establishes a classification system and risk model grounded in ARGs, offering valuable insights into the prognosis and treatment responsiveness of HCC.

## 1. Introduction

Hepatocellular carcinoma (HCC), the most common form of primary liver cancer, poses a significant global health challenge due to its high incidence, aggressive nature, and poor prognosis.^[1]^ According to the World Health Organization, HCC ranks as the sixth most prevalent cancer worldwide and the fourth leading cause of cancer-related mortality, with an estimated 905,677 new cases and 830,180 deaths in 2020 alone.^[2]^ The etiology of HCC is multifactorial, with chronic hepatitis B and C virus infections, alcohol abuse, nonalcoholic fatty liver disease, and exposure to aflatoxins being major risk factors.^[3]^ Despite advances in diagnostic techniques, surgical interventions, and targeted therapies, the overall survival rate for HCC patients remains unsatisfactory, largely due to late-stage diagnosis, frequent recurrence, and the development of drug resistance.^[4]^ This underscores the urgent need for a deeper understanding of the molecular mechanisms driving HCC progression and the identification of novel biomarkers that can predict patient outcomes and guide personalized therapeutic strategies.

Adenosine triphosphate (ATP) hydrolysis represents a fundamental energy-releasing process that fuels numerous cellular activities, including metabolism, signal transduction, and cytoskeletal dynamics.^[5]^ In addition to its role as the universal energy currency, ATP hydrolysis is tightly coupled to the function of numerous enzymes and molecular machines, such as kinases, guanosine triphosphateases, ion channels, and ATPases, which play critical roles in maintaining cellular homeostasis and regulating cell growth, differentiation, and death.^[6]^ Not surprisingly, dysregulation of ATP hydrolysis has been implicated in various diseases,^[7]^ particularly cancer,^[8,9]^ where it can contribute to metabolic reprogramming, oncogenic signaling, and the acquisition of invasive and metastatic phenotypes.^[8]^ Furthermore, several ATP-hydrolyzing proteins have been recognized as potential therapeutic targets or biomarkers in various malignancies,^[10,11]^ highlighting the importance of investigating their roles in HCC pathogenesis and progression.

This study aims to elucidate the role of ATP hydrolysis-related genes (ARG) in shaping the molecular landscape of HCC and to identify distinct molecular subtypes with unique clinical and prognostic characteristics. By leveraging large-scale genomic, transcriptomic, and clinical data from well-characterized HCC cohorts, we will systematically analyze the expression patterns, genetic alterations, and functional interactions of ATP hydrolysis-associated genes. We anticipate that this comprehensive approach will reveal novel insights into the biological pathways and processes influenced by ATP hydrolysis in HCC and help uncover potential therapeutic targets and prognostic biomarkers. Figure 1 presents a schematic overview of the workflow employed in the present study.

**Figure 1.**
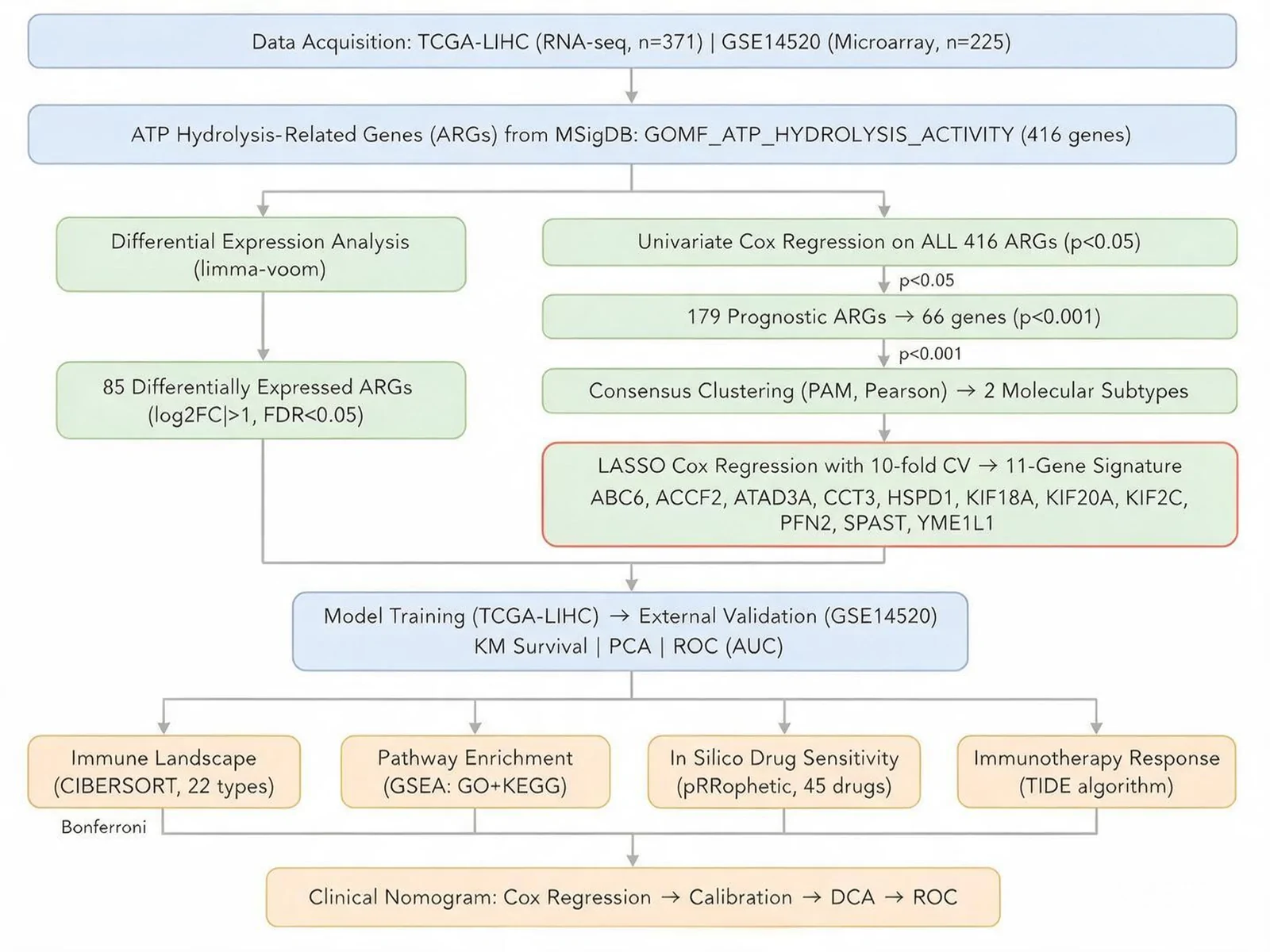
Workflow for identifying an ARG-derived risk signature in HCC. ARG = ATP hydrolysis-related gene, ATP = adenosine triphosphate, AUC = area under the curve, DCA = decision curve analysis, FDR = false discovery rate, GO = Gene Ontology, GSEA = gene set enrichment analysis, HCC = hepatocellular carcinoma, KEGG = Kyoto Encyclopedia of Genes and Genomes, KM = Kaplan–Meier, PAM = partitioning around medoids, PCA = principal component analysis, ROC = receiver operating characteristic, TIDE = tumor immune dysfunction and exclusion.

## 2. Materials and methods

### 2.1. Data acquisition

Transcriptomic, follow-up, and phenotype data for TCGA-LIHC cohort (liver hepatocellular carcinoma cohort/dataset from The Cancer Genome Atlas) were obtained from the University of California, Santa Cruz Xena platform (https://xenabrowser.net/datapages/) for model development. Following exclusion of cases with incomplete clinical-pathological information or follow-up periods shorter than 30 days, 371 HCC cases remained. For model validation, GSE14520 dataset was downloaded from the Gene Expression Omnibus (https://www.ncbi.nlm.nih.gov/gds/), comprising 225 HCC cases. The GOMF_ATP_HYDROLYSIS_ACTIVITY gene set containing 416 ARGs was retrieved from the Molecular Signatures Database (https://www.gsea-msigdb.org/gsea/msigdb/index.jsp). As the data were accessed from publicly available repositories, no additional ethical approval was required.

The GOMF_ATP_HYDROLYSIS_ACTIVITY gene set (Gene Ontology [GO]: 0016887) was selected because it represents the most direct and specific definition of ATP hydrolysis activity at the GO molecular function level, specifically capturing genes whose products catalyze ATP + H_2_O -> ADP + phosphate. This distinguishes it from broader categories such as ATP binding (GO: 0005524, >1500 genes). The 416 ARGs were retrieved from the Molecular Signatures Database via this gene set (Table S1, Supplemental Digital Content 1).

### 2.2. Differential expression analysis

Raw count data were normalized using transcripts per million method and subsequently log2-transformed. Differentially expressed ARGs were determined using the Limma package, with genes exhibiting absolute log2 fold change > 1 and adjusted *P* value < .05 considered significant.

### 2.3. Molecular subtyping

ARGs associated with HCC prognosis were assessed via univariate Cox regression analysis (*P* < .05). Consensus clustering was performed using the ConsensusClusterPlus package,^[12]^ employing the partitioning around medoids algorithm and Pearson distance metric, with 50 iterations and an 80% resampling rate. Kaplan–Meier (KM) survival curves and log-rank tests were used to evaluate differences in prognosis among identified subtypes, complemented by principal component analysis (PCA).

### 2.4. Construction of prognostic risk signature

ARGs with *P* < .001 in the prognostic analysis underwent least absolute shrinkage and selection operator (LASSO) Cox regression analysis with type.measure set to “deviance” and 1000 iterations. The risk score formula was defined as: riskscore = Σ(β_i × exp_level_i), where β_i denotes the coefficient of gene i, and exp_level_i represents the expression level of gene i. High- and low-risk groups were established based on the median riskscore in both training and validation sets, with KM survival curves, log-rank tests, PCA, and receiver operating characteristic (ROC) curve analyses used to assess differences in prognosis.

ARGs demonstrating strong prognostic association (*P* < .001) were selected to balance variable reduction with model feasibility. LASSO Cox regression with 10-fold cross-validation was then applied to construct the final prognostic signature.

### 2.5. Immune landscape and treatment response assessment

The IOBR package (an R package for tumor immunology and immunotherapy analysis)^[13]^ was utilized to evaluate the immune landscape of HCC patients, with Cell-type Identification By Estimating Relative Subsets Of RNA Transcripts employed to quantify infiltration levels of 22 immune cell types. Drug sensitivity was evaluated using the pRRophetic package (14) for in silico prediction of clinical chemotherapeutic response.^[14]^ The tumor immune dysfunction and exclusion (TIDE) algorithm was applied to assess the potential response to immunotherapy.^[15]^

### 2.6. Gene set enrichment analyses (GSEA) and mutation analysis

GO annotations and Kyoto Encyclopedia of Genes and Genomes pathway GSEA were performed using the clusterProfiler package,^[16]^ while somatic mutation analysis and visualization were carried out using maftools.

### 2.7. Building and validating a predictive nomogram

Univariate and multivariate Cox regression analyses were conducted to evaluate HCC prognostic factors. A nomogram was constructed using the rms package, with calibration curves, ROC curves, and decision curve analysis (rmda package) employed to assess its performance and compare its prognostic accuracy to other established factors.

### 2.8. Statistical analysis

Statistical analyses were conducted using R software version 4.3.2 (R Foundation for Statistical Computing). Nonparametric Wilcoxon rank-sum tests were used for intergroup comparisons, with statistical significance set at *P* < .05.

## 3. Results

### 3.1. Prognostic signatures and molecular subtyping related to ARGs

Differential expression analysis revealed that 85 ARGs were aberrantly expressed in HCC, with 41 overexpressed and 44 underexpressed (Fig. 2A). Univariate Cox regression analysis identified 179 ARGs significantly associated with HCC prognosis (*P* < .05; Table S2, Supplemental Digital Content 2). Among these, genes belonging to the ATP binding cassette subfamily A and dynein axonemal heavy chain were prominently mutated (Fig. 2B). Consensus clustering based on these prognosis-related ARGs defined 2 molecular subtypes (Fig. 2C), which showed statistically significant differences in survival (Fig. 2D). PCA of the same prognostic ARGs is presented in Figure 2E.

**Figure 2.**
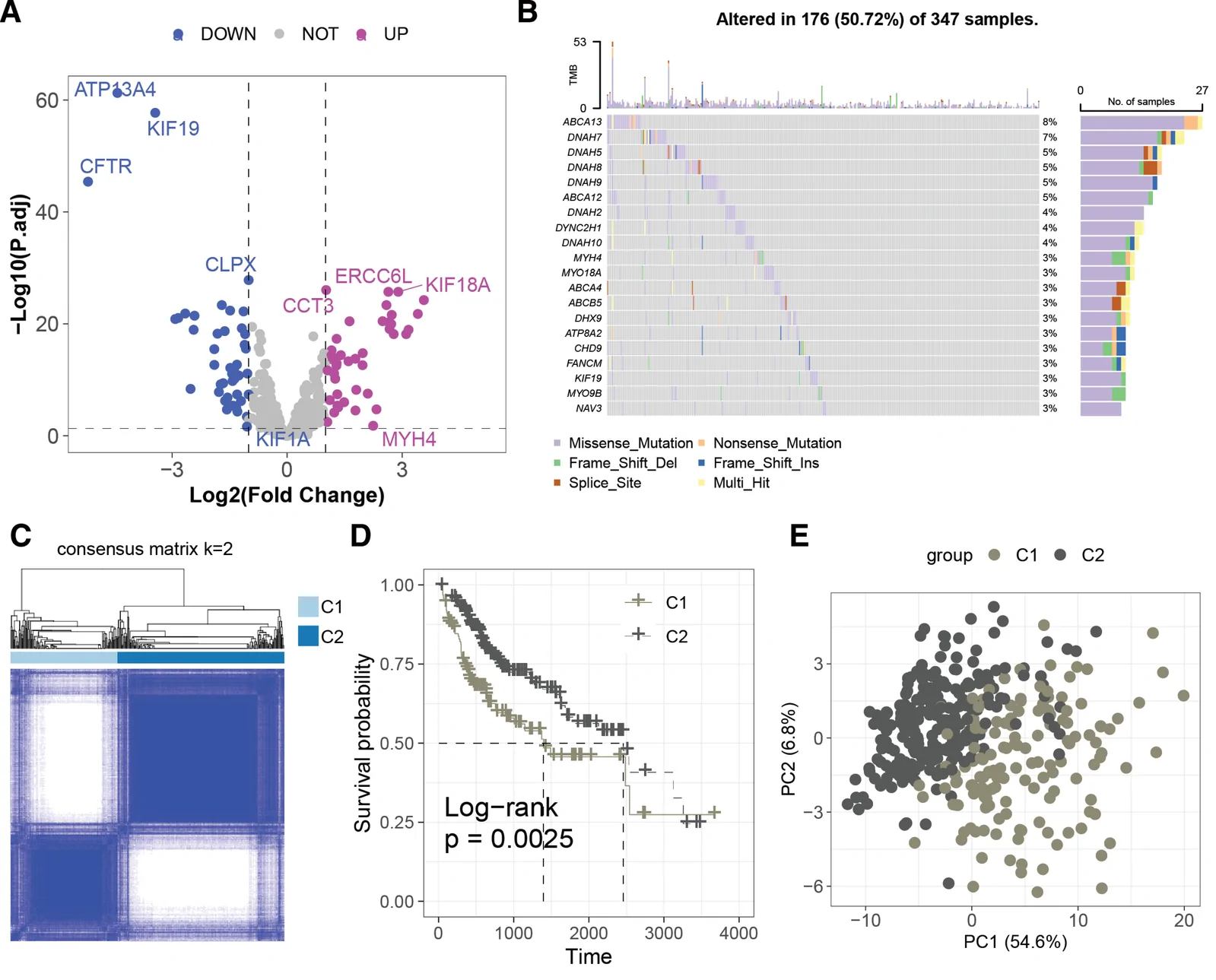
ARGs in prognosis-related context and their involvement in HCC molecular subtyping. (A) Volcano plot depicting differentially expressed ARGs between cancer and adjacent normal tissues in the TCGA-LIHC cohort, (B) somatic mutation landscape of ARGs in the TCGA-LIHC cohort, (C) consensus clustering based on ARGs with prognostic significance, (D) KM survival analysis of C1 and C2 subtypes, (E) PCA of ARGs associated with prognosis. ARG = ATP hydrolysis-related gene, ATP = adenosine triphosphate, HCC = hepatocellular carcinoma, KM = Kaplan–Meier, PCA = principal component analysis, TCGA-LIHC = liver hepatocellular carcinoma cohort/dataset from The Cancer Genome Atlas.

### 3.2. Construction and evaluation of a prognostic signature derived from ARGs

Of the 179 prognosis-related ARGs, 66 with *P* < .001 were subjected to LASSO Cox regression analysis (Figs. 3A and 3B). This resulted in an 11-gene risk signature (Fig. 3C): riskscore = 0.198 * YME1L1 + 0.048 * SPAST + 0.088 * PFN2 + 0.052 * KIF2C + 0.105 * KIF20A + 0.062 * KIF18A + 0.173 * HSPD1 + 0.106 * CCT3 + 0.225 * ATAD3A + 0.092 * ABCF2 + 0.839 * ABCB6. Patients in the TCGA-LIHC and GSE14520 cohorts were dichotomized into high- and low-risk groups based on the median riskscore. KM survival analysis demonstrated significantly poorer prognosis in the high-risk group compared to the low-risk group for both cohorts (Figs. 3D–3G). PCA clearly discriminated between high- and low-risk groups (Figs. 3E–3H). ROC curves demonstrated areas under the curve (AUCs) of 0.826, 0.717, and 0.657 for 1-, 3-, and 5-year overall survival prediction in the TCGA-LIHC cohort (Fig. 3F), and AUCs of 0.608, 0.603, and 0.621 for the corresponding survival times in the GSE14520 cohort (Fig. 3I).

**Figure 3.**
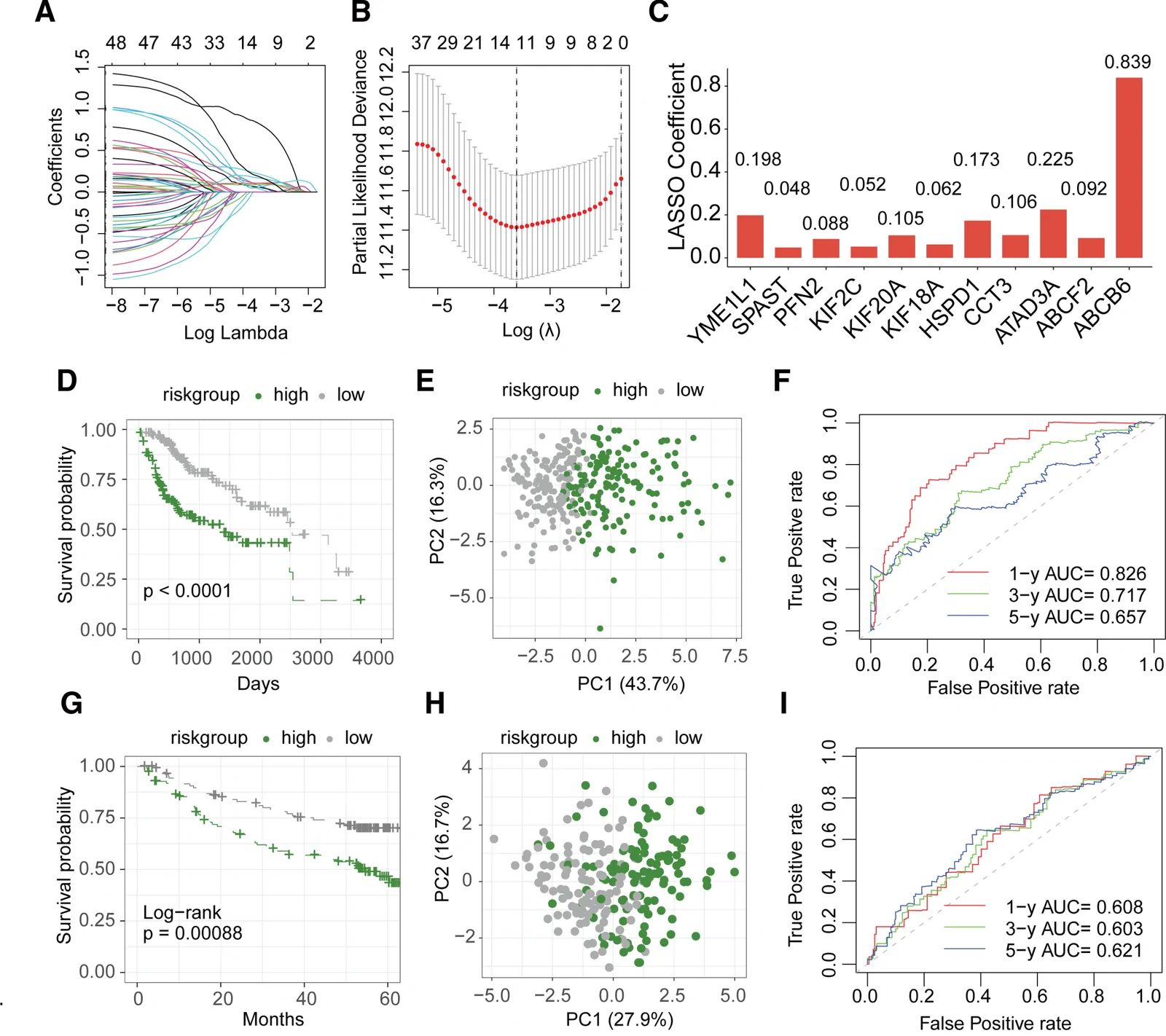
Construction of an HCC prognosis signature based on ARGs. (A, B) LASSO Cox regression analysis, (C) coefficients of genes composing the riskscore. KM survival analysis, PCA, and ROC curve analysis for the TCGA-LIHC cohort (D, E, F) and the GSE14520 cohort (G, H, I). ARG = ATP hydrolysis-related gene, ATP = adenosine triphosphate, HCC = hepatocellular carcinoma, KM = Kaplan–Meier, LASSO = least absolute shrinkage and selection operator, PCA = principal component analysis, ROC = receiver operating characteristic, TCGA-LIHC = liver hepatocellular carcinoma cohort/dataset from The Cancer Genome Atlas.

In the training cohort, the median risk score was 3.85, which served as the threshold for group stratification. Due to platform differences between RNA-seq (TCGA) and microarray (GSE14520) data, the median risk score in the validation cohort was 10.59, highlighting the critical role of platform-specific normalization and the importance of dataset-specific cutoff determination.

### 3.3. Clinical and pathological landscape of the ARG-derived signature

Heat map analysis of the expression levels of genes contributing to the risk signature (Fig. 4A) revealed a consistent and significant upregulation in high-risk group samples, suggesting a co-expression pattern potentially associated with disease severity and pathophysiological roles in high-risk individuals. Investigating the relationship between the riskscore and clinical parameters, we found a significant association with survival status and pathological staging (Fig. 4B). Notably, deceased patients had significantly higher riskscores compared to survivors, indicating a negative correlation between riskscore and survival outcome. Moreover, advanced T stages, overall stages, and high-grade tumors exhibited elevated riskscores relative to their early-stage and low-grade counterparts, reinforcing the signature’s potential as a biomarker reflecting disease progression and malignancy.

**Figure 4.**
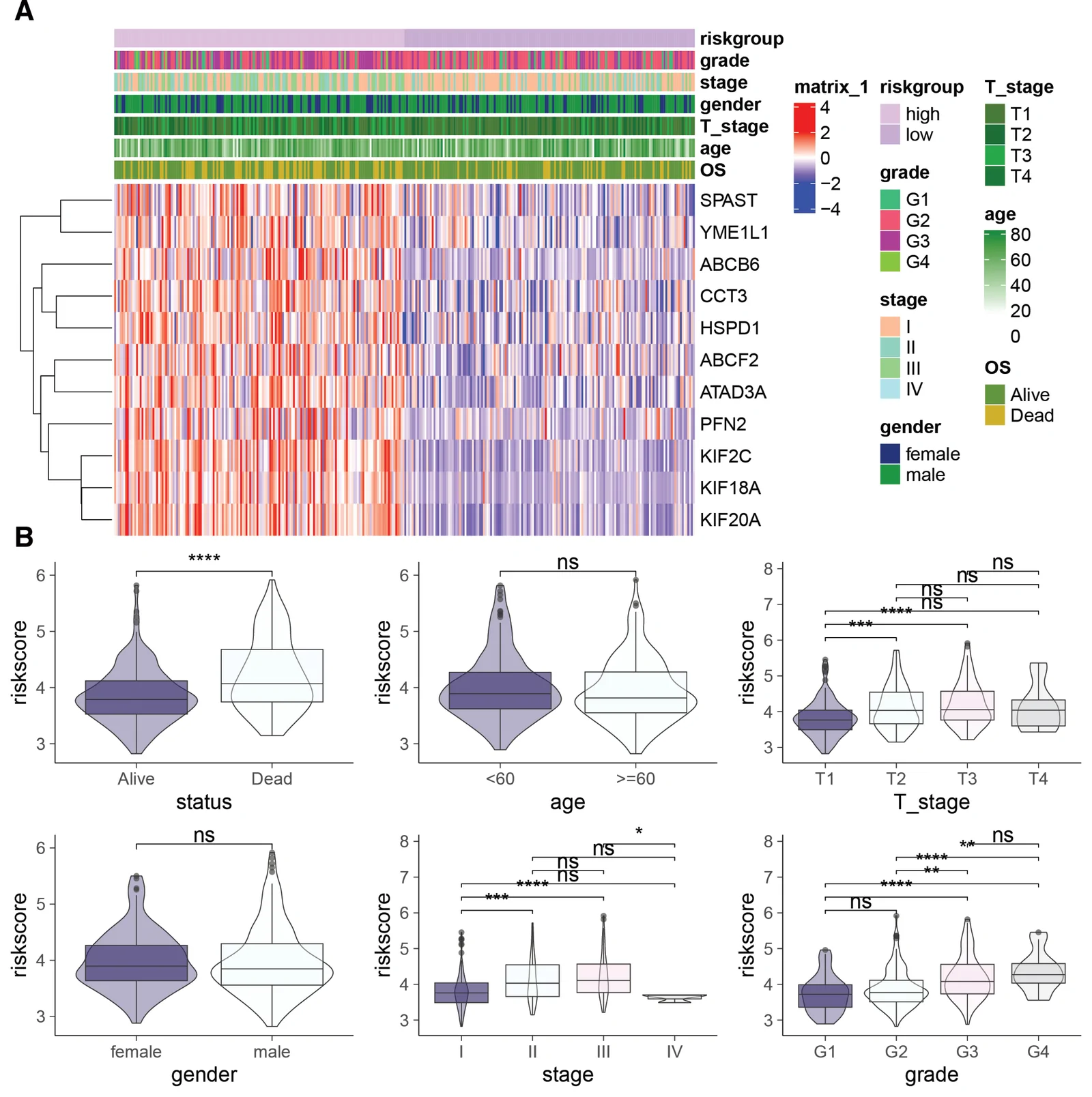
Clinical and pathological distribution of the ARG-derived HCC risk signature. (A) Heatmap of gene expression and clinical-pathological annotation for genes contributing to the riskscore, (B) comparison of riskscores across different clinical-pathological subgroups. ARG = ATP hydrolysis-related gene, ATP = adenosine triphosphate, HCC = hepatocellular carcinoma, Ns = not significant. *P < .05, **P < .01, ***P < .001, ****P < .0001.

Gene expression and somatic mutations associated with the ARG-derived signature. GSEA indicated that catabolic processes, oxidoreductase activity, and monooxygenase activity were significantly activated in the low-risk group compared to the high-risk group, whereas chromatid segregation and mitotic nuclear division were suppressed (Fig. 5A). Additionally, the low-risk group showed enhanced activation of complement and coagulation cascades and drug metabolism pathways, while spliceosome, DNA replication, and cell cycle pathways were downregulated (Fig. 5B). The top 10 most frequently mutated genes in the high-risk group were TP53, CTNNB1, TTN, MUC16, LRP1B, APOB, OBSCN, RYR2, XIRP2, and ALB (Fig. 5C), whereas those in the low-risk group were CTNNB1, TTN, ALB, MUC16, PCLO, TP53, RYR2, SPTA1, APOB, and AXIN1 (Fig. 5D).

**Figure 5.**
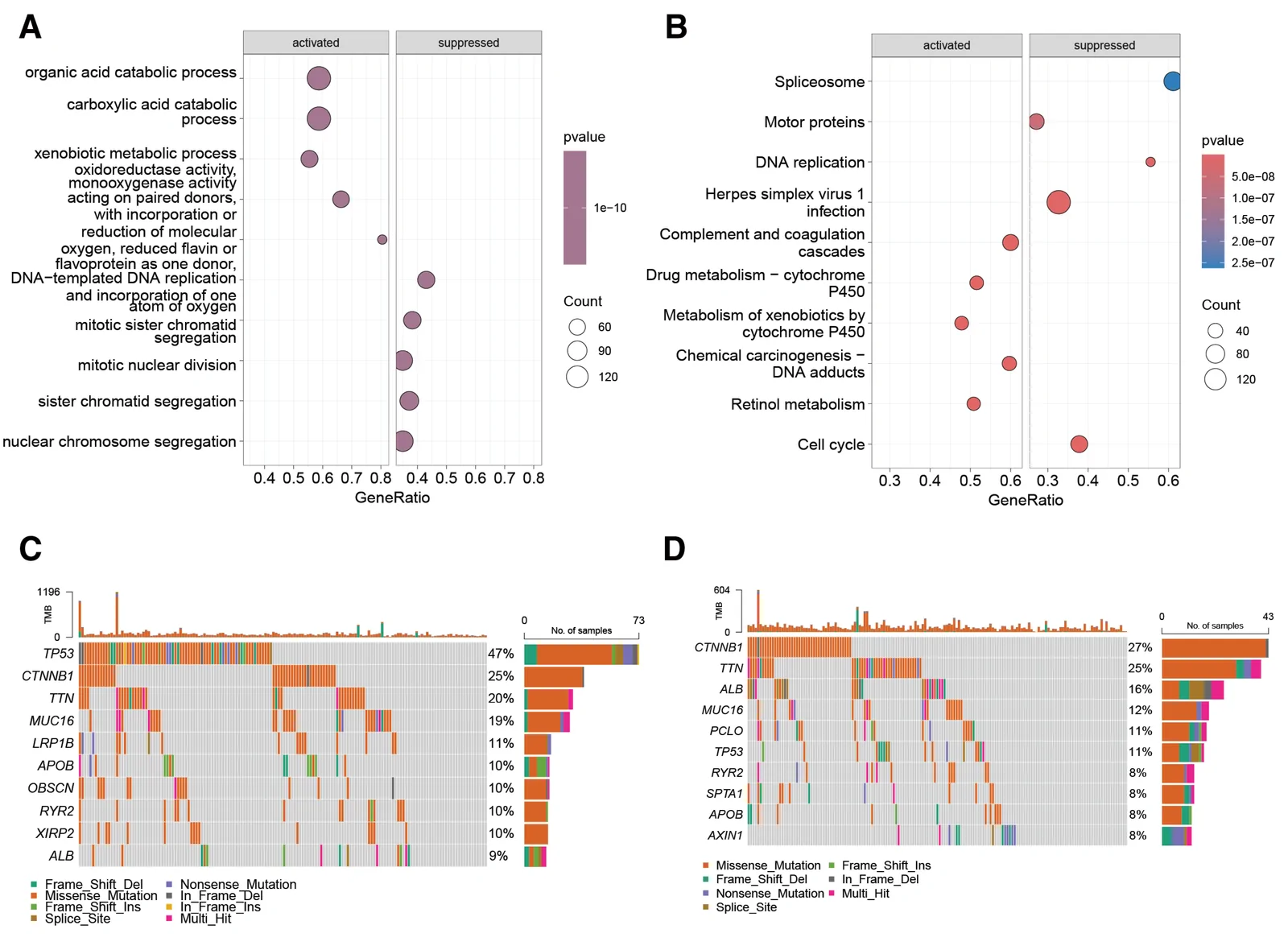
Gene expression and mutation patterns related to the ARG-derived risk signature in HCC. (A) GO GSEA, (B) KEGG pathway gene set enrichment analysis, (C) oncoplot showing somatic mutations in genes from the high-risk group, (D) oncoplot displaying somatic mutations in genes from the low-risk group. ARG = ATP hydrolysis-related gene, ATP = adenosine triphosphate, GO = Gene Ontology, GSEA = gene set enrichment analysis, HCC = hepatocellular carcinoma, KEGG = Kyoto Encyclopedia of Genes and Genomes.

### 3.4. Association of the ARG-derived risk signature with the immune landscape and immune therapy response

Immune infiltration analysis revealed increased infiltration of macrophages, follicular helper T cells, and activated memory CD4 T cells, along with decreased infiltration of B cells, mast cells, monocytes, and resting memory CD4 T cells in the high-risk group compared to the low-risk group (Fig. 6A). Correlation analysis indicated that resting CD4 + T cells, B cells, and resting mast cells were negatively correlated with the expression of most risk signature genes, whereas macrophages and activated CD4 + T cells showed positive correlations (Fig. 6B). Furthermore, the high-risk group had significantly higher TIDE scores (Fig. 6C), suggesting computationally inferred inferior responsiveness to immune checkpoint blockade therapy. After Bonferroni correction for multiple testing across 22 immune cell types, 6 of 8 nominally significant cell types remained significant: Macrophages M0 (adjusted *P* = 9.0 × 10 − 6), CD4 memory resting T cells (*P* = 1.4e−5), Monocytes (*P* = 3.8e−5), Follicular helper T cells (*P* = .009), Resting mast cells (*P* = .028), and Activated CD4 memory T cells (*P* = .044). Complete adjusted *P* values are reported in Table S3, Supplemental Digital Content 3.

**Figure 6.**
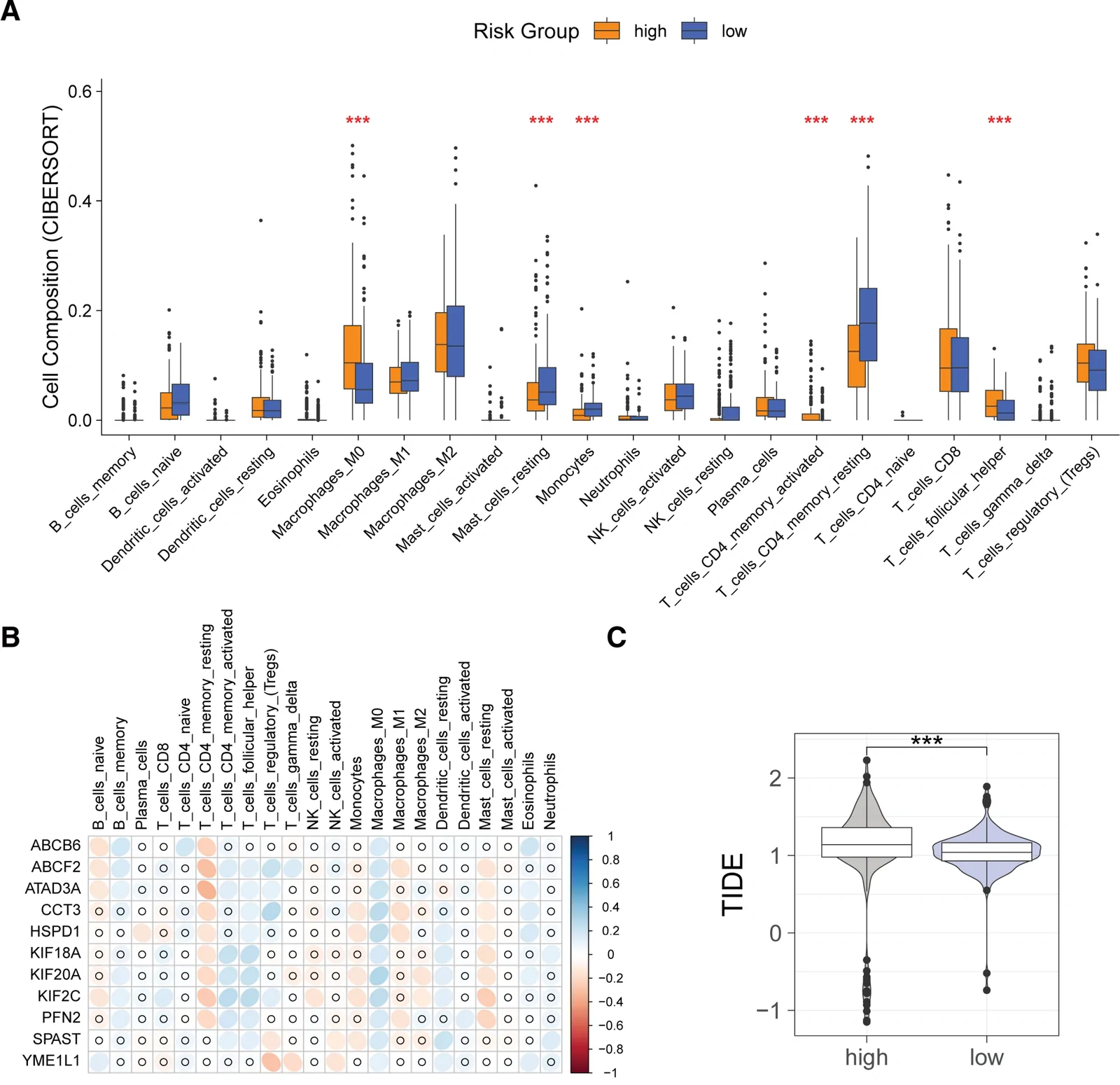
Relationship between the ARG-derived risk signature and the immune landscape in HCC. (A) Comparison of immune cell infiltration levels between high- and low-risk groups. ***P < .05 after Bonferroni correction across 22 immune cell types; (B) Heatmap depicting correlations between riskscore component genes’ expression and immune cell infiltration. *P < .05, **P < .01, ***P < .001 after Bonferroni correction. (C) Comparison of TIDE scores between high- and low-risk groups. ARG = ATP hydrolysis-related gene, ATP = adenosine triphosphate, HCC = hepatocellular carcinoma, TIDE = tumor immune dysfunction and exclusion.

### 3.5. Indication of drug sensitivity by the ARG-derived risk signature

Drug sensitivity analysis demonstrated significant differences in sensitivity to 29 drugs (Bonferroni-corrected *P* < .05) between high- and low-risk groups (Fig. 7A, Table S4, Supplemental Digital Content 4). Most ARGs were significantly correlated with drug sensitivity, and many genes displayed consistent correlations for the same drug, implying a crucial role for ATP hydrolysis in drug metabolism (Fig. 7B).

**Figure 7.**
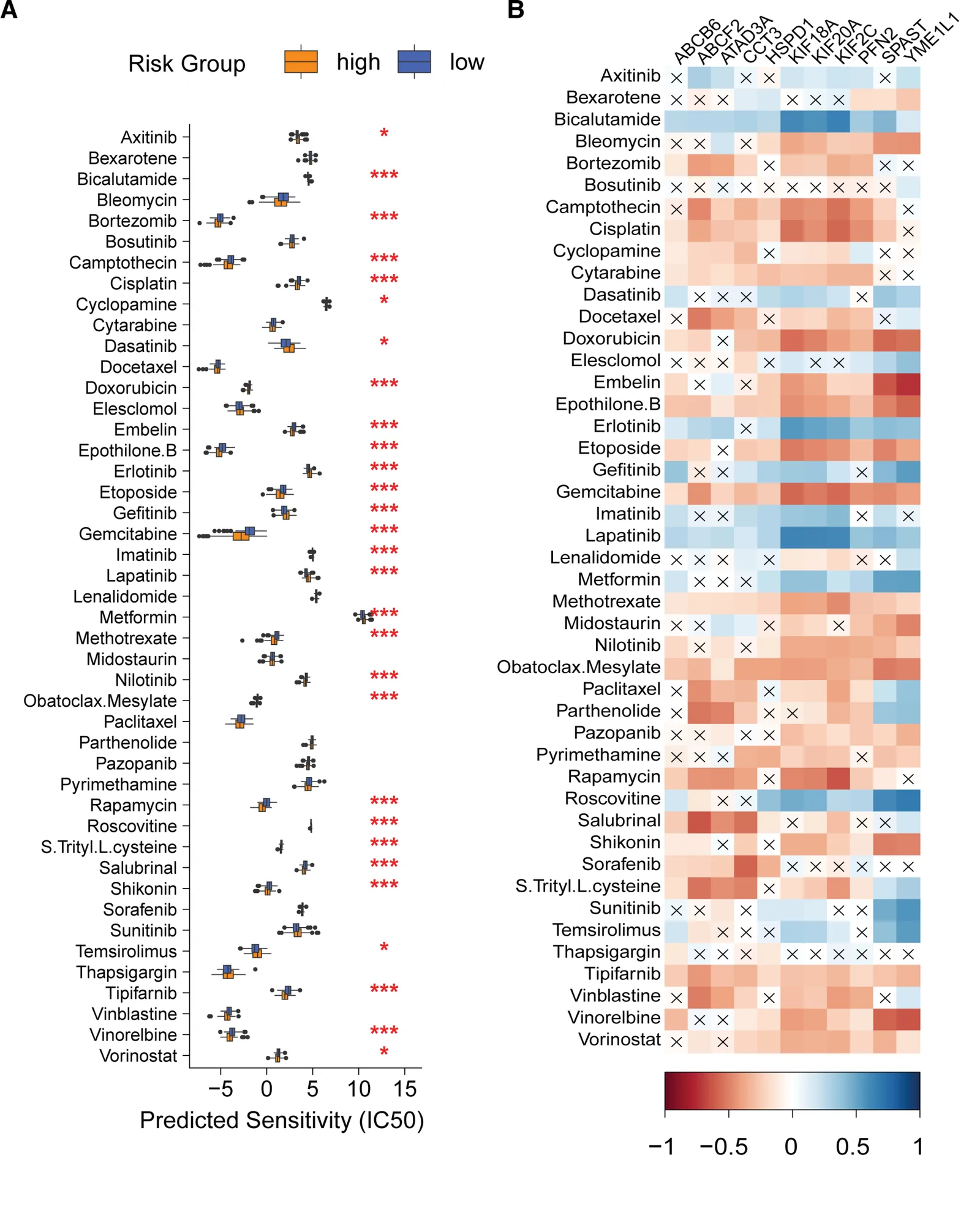
In silico prediction of drug sensitivity using the ARG-derived HCC risk signature. (A) Comparison of drug sensitivity differences between high- and low-risk groups. ***P < .05 after Bonferroni correction across 45 drugs. (B) Heatmap showing correlations between riskscore component gene expression and drug sensitivity. *P < .05, **P < .01, ***P < .001 after Bonferroni correction. ARG = ATP hydrolysis-related gene, ATP = adenosine triphosphate, HCC = hepatocellular carcinoma.

### 3.6. Nomogram based on the ARG-derived risk signature

Univariate Cox regression identified risk score, T stage, and overall stage as prognostic factors for HCC, but only the risk score retained significance in multivariate analysis (Figs. 8A and 8B). Consequently, a nomogram was constructed using the risk score to predict 1-, 3-, and 5-year overall survival (Fig. 8C). The calibration curve showed close agreement between observed and nomogram-predicted overall survival, indicating good predictive performance (Fig. 8D). Decision curves for the nomogram and individual clinical factors in predicting 1-year overall survival are shown in Figure 8E. The ROC curve demonstrated AUCs of 0.807, 0.69, and 0.63 for 1-, 3-, and 5-year survival prediction, respectively (Fig. 8F).

**Figure 8.**
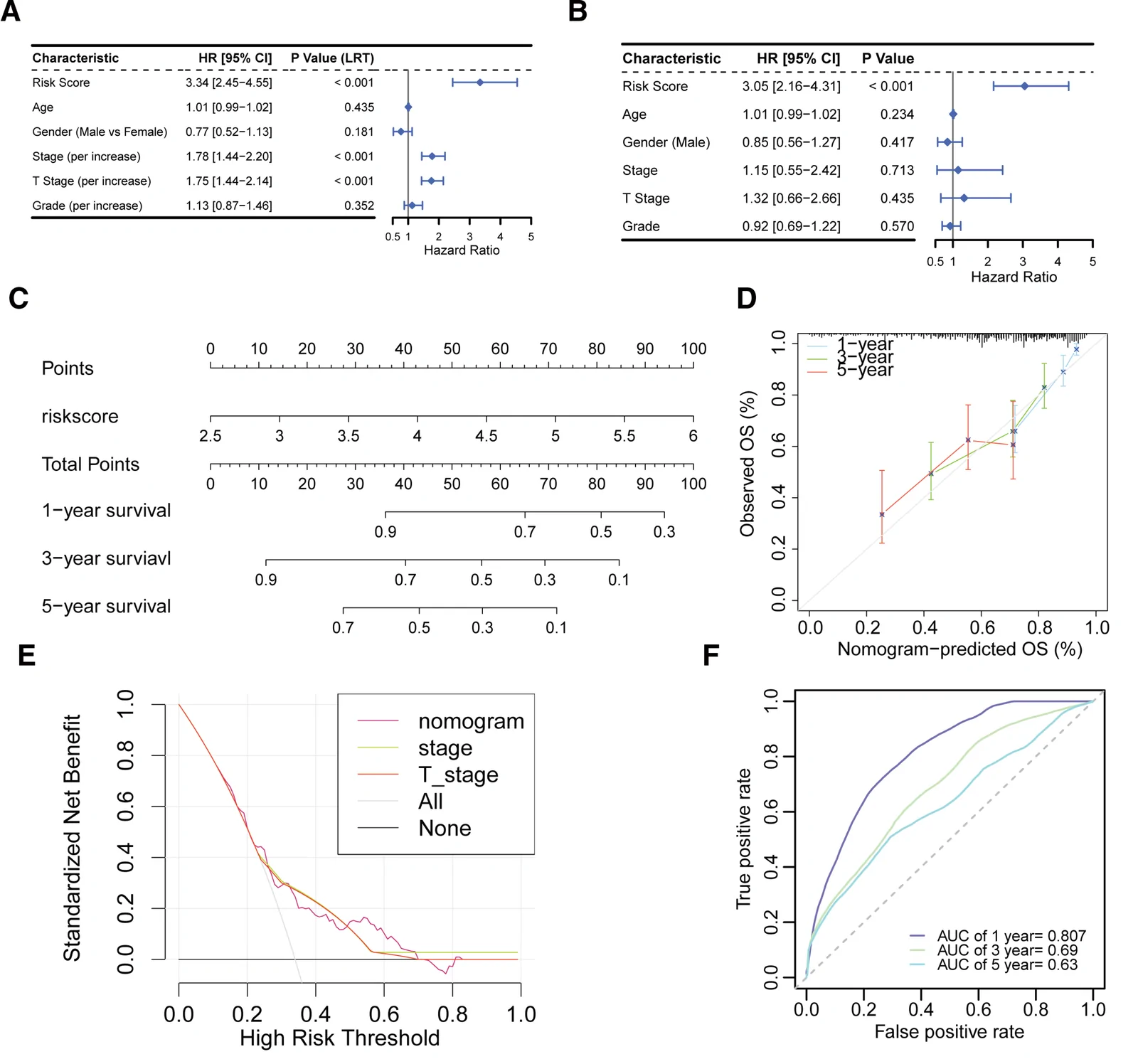
Development of a nomogram for HCC prognosis prediction based on the ARG-derived risk signature. (A) Univariate and (B) multivariate Cox regression analyses (*P* values from Likelihood Ratio Test for multilevel factors), (C) nomogram constructed using the riskscore for predicting 1-, 3-, and 5-year overall survival, (D) calibration curve for the nomogram, (E) DCA comparing the nomogram with individual clinical factors, (F) ROC curves for the nomogram at 1-, 3-, and 5-year survival prediction. ARG = ATP hydrolysis-related gene, ATP = adenosine triphosphate, DCA = decision curve analysis, HCC = hepatocellular carcinoma, ROC = receiver operating characteristic.

## 4. Discussion

HCC continues to pose a significant global public health challenge.^[1]^ Traditional parameters such as TNM staging, vascular invasion, and AFP levels offer some predictive value for HCC prognosis, yet the heterogeneity of this malignancy necessitates the identification of novel prognostic biomarkers and the development of more precise predictive models.^[17]^ The integration of multi-gene prognostic signatures with conventional clinical parameters offers a promising avenue for enhanced cancer prognosis assessment.^[18]^

This study employed ARG analysis to discern molecular subtypes of HCC and derived a novel prognostic feature that robustly predicts patient outcomes and therapeutic responses. This feature encompasses an 11-gene signature comprising YME1L1, SPAST, PFN2, KIF2C, KIF20A, KIF18A, HSPD1, CCT3, ATAD3A, ABCF2, and ABCB6.

YME1L1, an inner mitochondrial protease involved in mitochondrial homeostasis and quality control, exhibits dual tumor-suppressive (metabolic regulation, apoptosis induction) and oncogenic potential in cancer. PFN2, a component of the γ-secretase complex, participates in the cleavage of transmembrane proteins like Notch and APP and may influence prognosis through modulation of immune infiltrates in glioma patients.^[19]^ Kinesins KIF2C, KIF20A, and KIF18A, frequently overexpressed in HCC and other tumors, are associated with proliferation, invasiveness, metastasis, and poor prognosis.^[20–22]^ HSPD1, encoding heat shock protein Hsp60, is implicated in protein folding, cellular homeostasis, and stress response, with its overexpression in HCC linked to tumorigenesis, invasiveness, and drug resistance.^[23,24]^ CCT3 encodes the TRiC complex subunit CCTγ, participating in protein folding; its dysregulation may impact the stability and function of key tumor proteins.^[25,26]^ Collectively, these findings substantiate the potential utility of ARG-based biomarkers in HCC.

ATP hydrolysis plays multiple roles in tumor immunology: serving as a “danger signal” for inflammation and immune activation, triggering DCs, macrophages, and NK cell activation^[27,28]^; participating in the regulation of immune checkpoints like PD-L1^[29]^; providing energy for effector immune cells; and generating adenosine via A2A/A2B receptors, which induces immunosuppression.^[30]^ Aberrant expression or activity changes of ARG can influence tumor metabolism, proliferation, and migration, indirectly shaping an immunosuppressive microenvironment or directly modulating immune cell functions. Targeting these genes or their encoded enzymes, e.g., inhibiting adenosine production via CD39/CD73 blockade,^[31]^ or interfering with tumor metabolism, could potentiate immunotherapy efficacy.^[32]^ The ARG-based HCC prognostic signature revealed associations with patient immune status and prognosis, offering a fresh perspective on personalized therapy. These mechanistic connections warrant further experimental investigation to validate the specific roles of individual signature genes in HCC immune regulation.

ARGs are closely tied to HCC prognosis and drug sensitivity. Their abnormal expression or functional perturbations can affect tumor metabolism (e.g., Warburg effect), indicating sensitivity to metabolic inhibitors^[33]^; regulate drug transporter activities, influencing drug uptake and efflux; alter drug targets (e.g., kinases) interaction with ATP, impacting treatment efficacy^[34]^; and participate in drug resistance mechanisms (e.g., upregulating efflux pumps, modifying metabolic enzyme activities).^[35]^ The ARG-derived prognostic signature reflects tumor metabolic status, drug handling capacity, and signaling complexity, correlating with the effectiveness of specific treatment strategies, thus holding promise for guiding individualized therapy. Further research aims to elucidate underlying molecular mechanisms and enhance clinical applicability. While these computational predictions provide a foundation for further investigation, experimental validation is essential to confirm the identified associations.

Regarding clinical applicability, the nomogram developed in this study serves as a proof-of-concept tool that integrates the ARG-derived risk signature with clinical features to provide individualized prognostic estimates. However, several steps would be required before clinical implementation: prospective validation in multi-center cohorts with standardized sample processing protocols; development of a clinically feasible assay (e.g., targeted quantitative polymerase chain reaction panel) for the 11-gene signature; establishment of platform-independent cutoff values through cross-platform calibration; and demonstration of clinical utility through decision-impact studies showing that the nomogram alters clinical management or improves patient outcomes. At present, the nomogram should be considered a research tool that generates hypotheses rather than a ready-to-use clinical decision instrument.

While this study provides valuable new insights into HCC prognosis and treatment responsiveness, several limitations should be acknowledged. Firstly, despite utilizing large-scale datasets from multiple databases, sample heterogeneity may limit the generalizability of certain observations. Secondly, although the ARG risk feature associates with diverse immune cell infiltrates and drug sensitivities, causal relationships and specific molecular mechanisms underlying these associations require further experimental validation. Lastly, while the constructed nomogram demonstrated favorable predictive performance in predicting HCC patient prognosis, its practical value in clinical practice remains to be validated by prospective cohort studies. Future research should consider integrating diverse molecular data types to enhance the comprehensiveness and accuracy of predictive models.

Firstly, the retrospective nature of publicly available datasets introduces inherent selection biases that may affect generalizability. Secondly, substantial batch effects exist between different measurement platforms (RNA-seq vs microarray), as evidenced by the risk score distribution differences observed (training median = 3.85 vs validation median = 10.59), underscoring the need for cross-platform normalization methods. Thirdly, the identified gene signature and computationally predicted drug sensitivities lack experimental validation through in vitro functional assays and in vivo models. Fourthly, incomplete clinical variable coverage in TCGA (including absence of AFP levels, vascular invasion status, treatment history, and liver function parameters) limits comprehensive clinical modeling. Fifthly, both drug sensitivity predictions (pRRophetic) and immunotherapy response predictions (TIDE) are entirely in silico and should be interpreted as hypothesis-generating rather than definitive. Sixthly, external validation was performed in only one independent cohort (GSE14520); multi-cohort, multi-platform validation is needed to confirm the robustness of the signature.

## 5. Conclusion

In conclusion, this study systematically elucidated the association between ARG and HCC prognosis, identifying 2 distinct ARG-derived HCC molecular subtypes and constructing and evaluating an 11-gene ARG-derived risk signature that effectively predicts HCC patient outcomes, drug responses, and responses to immunotherapy. However, the precise mechanistic underpinnings of ARG’s involvement in HCC pathogenesis, tumor immune microenvironment modulation, and drug responsiveness remain to be deciphered through further biological experimentation. Moreover, the prognostic utility of the ARG-derived signature warrants validation in prospective clinical studies.

## Author contributions

**Conceptualization:** Yan Dong, Feng Yu.

**Data curation:** Yan Dong, Feng Yu.

**Formal analysis:** Yan Dong, Feng Yu.

**Funding acquisition:** Yan Dong, Feng Yu.

**Investigation:** Yan Dong, Feng Yu.

**Methodology:** Yan Dong, Feng Yu.

**Project administration:** Yan Dong, Feng Yu.

**Resources:** Yan Dong, Feng Yu.

**Software:** Yan Dong, Feng Yu.

**Supervision:** Yan Dong, Feng Yu.

**Validation:** Yan Dong, Feng Yu.

**Visualization:** Yan Dong, Feng Yu.

**Writing – original draft:** Yan Dong, Feng Yu.

**Writing – review & editing:** Yan Dong, Feng Yu.








